# The determination of pH of peri‐implant crevicular fluid around one‐piece and two‐piece dental implants: A pilot study

**DOI:** 10.1002/cre2.177

**Published:** 2019-03-13

**Authors:** Dainius Karpavicius, Morta Stasikelyte, Nomeda Baseviciene, Urte Sakalauskaite, Saule Ratkute, Dainius Razukevicius

**Affiliations:** ^1^ Department of Prosthetic Treatment and Implantology of Maxillofacial Clinic Lithuanian University of Health Sciences Kaunas Lithuania; ^2^ Department of Periodontology Lithuanian University of Health Sciences Kaunas Lithuania; ^3^ Department of Plastic and Reconstructive Surgery Lithuanian University of Health Sciences Kaunas Lithuania

**Keywords:** dental implant design, gingival crevicular fluid, gingival sulcus, peri‐implant sulcus, peri‐implant tissues

## Abstract

There are two main groups of screw‐type dental implant designs: one‐piece and two‐piece implants. Although success rates of both of these types of implants are high, none of them avoid complications, of which the most common are peri‐implant mucositis and peri‐implantitis. Current clinical diagnostic parameters are relatively noninvasive and cost‐efficient; however, they are often not sensitive enough and fail to determine the activity of inflammation. The purpose of this study is to determine pH of peri‐implant crevicular fluid (PICF) around one‐piece and two‐piece implants and pH of gingival crevicular fluid (GCF) around healthy teeth and to find out if our suggested method could function accurately for determination of pH of PICF and GCF. Thermo Fisher Scientific™ Orion™ 9863BN glass microelectrode was used to determine pH of PICF around 29 one‐piece (ROOTT, TRATE AG) and 29 two‐piece implants (multiple manufacturers) and pH of GCF around 29 healthy teeth. pH of PICF around two‐piece implants was more acidic (*P* < 0.001). Average pH around one‐piece implants was 6.46 and around two‐piece implants was 6.15. Mean pH of GCF was 6.64. pH of PICF in women around two‐piece implants was more alkaline (*P* < 0.05); no difference was found in control and one‐piece implant groups. There was no statistically significant correlation found between age and pH of PICF and GFC. Design of dental implants has an impact on pH of PICF. Glass microelectrode is an appropriate tool for accurate determination of pH in PICF.

## 1 INTRODUCTION

Dental implants have been widely used as one of the most effective and long‐lasting rehabilitation methods for missing teeth over the last 60 years (Jepsen et al., 2015). During that time, they have been constantly changing and improving, and variety of their designs as well as surgical methods have significantly increased. Nowadays, many different types of dental implants differing in implant surface modifications, materials, surgical protocols, sizes, and designs are provided in the market. Among different screw‐type dental implant designs, there are two main types distinguished: one‐piece and two‐piece dental implants (Gamper et al., 2017). One‐piece dental implant has a rough intraosseous part and a smooth neck together functioning as a single solid unit, whereas two‐piece implants consist of an abutment that is attached to the implant body through a screw joint (Scacchi, Merz, & Schär, 2000). Although scientific studies show a high success rate of dental implants—even 96.8% survival rate in 5‐year period (Jepsen et al., 2015)—they still do not avoid complications (Jung et al., 2008; Pjetursson et al., 2004). Recently, the long‐stage complications of dental implants have been widely discussed. Peri‐implant mucositis is described as an inflammation of soft tissues around dental implant without any signs of marginal bone resorption. This condition is reversible. Although in case of peri‐implantitis in addition to inflammation of soft tissues, more than 2 mm of bone resorption is observed (Lang & Berglundh, 2011); the prevalence of peri‐implant mucositis and peri‐implantitis is 43% and 22%, respectively (Lang & Berglundh, 2011). Insufficient personal oral hygiene, smoking, type 2 diabetes mellitus, and history of chronic periodontitis are indicated as risk factors of these peri‐implant diseases (Lindhe, Meyle, and Group D of the European Workshop on Periodontology, 2008). In addition to these factors, the influence of implant design is being more often discussed as a possible risk factor.

The diagnostics of peri‐implant mucositis and peri‐implantitis is getting more and more important in implant dentistry, because it can cause no symptoms until the inflammation process is well advanced. This is the reason why it is essential to identify a disease early enough to prevent it from further progression. Current diagnostic methods and parameters were introduced more than 50 years ago and still are considered as a gold standard in periodontology and implant dentistry. These are probing pocket depth, bleeding on probing, clinical attachment level, plaque indexes, and radiologic evaluation of marginal bone level. Even though these diagnostic parameters are cost‐efficient and relatively noninvasive, however, they lack of sensitivity, because most of them are able to detect a progression of inflammation only if a change of 2 or 3 mm occurs in pocket probing depth or marginal bone level in radiograph. For example, conventional radiography is not sensitive enough and fail to show buccal and lingual bone defects (Kinney, Ramseier, & Giannobilea, 2007). Moreover, these methods fail to indicate the activity of inflammation. Because of these reasons, there is a need for new noninvasive, cost‐efficient, and sensitive diagnostic methods, which would be able to detect inflammation in early stages, show smaller changes than previously mentioned methods, and unable to tell if inflammation is in active or stabilized phase.

Nowadays, a lot of attention is being brought to investigation of oral fluids (saliva, gingival crevicular fluid [GCF], and peri‐implant crevicular fluid [PICF]) in order to find out if examination of these fluids could become a new routine method for diagnosis of peri‐implant mucositis and peri‐implantitis (Castagnola et al., 2011; Kinney et al., 2007). Investigations of PICF seem to be particularly promising. Bevilacqua, De Biasi, Lorenzon, Frattini, and Angerame (2016) discovered that flow of PICF increases if inflammation of peri‐implant tissues is present and that its volume differs in case of peri‐implant mucositis and peri‐implantitis. Other authors focused on biologic markers in PICF and on their qualitative and quantitative changes during peri‐implant tissues inflammation (Basegmez, Yalcin, Yalcin, Ersanli, & Mijiritsky, 2012; Ma et al., 2000; Nomura et al., 2000; Ramseier et al., 2016). Also, determination of pH of PICF was introduced as a promising diagnostic method. However, there has been only one scientific study performed by Nyako, Watson, and Preston (2005), whose aim was to determine pH values of PICF. Authors found out that there is a statistically significant difference between pH of PICF around healthy and failing dental implants. However, due to a small sample size, final conclusions cannot be made.

## 2 MATERIALS AND METHODS

### 2.1 Ethical considerations

The study was designed as a pilot clinical study. Following approval by the Ethical Committee of Lithuanian University of Health Sciences (TPP‐4470), 27 patients (16 women and 11 men), who had a dental implant therapy performed at the Department of Prosthetic Treatment and Implantology of Maxillofacial Clinic at Lithuanian University of Health Sciences, were selected for the study.

### 2.2 Participants

The selected patients had been treated with both one‐piece and two‐piece dental implants in period of 3–12 months. All the implants, included in the study, were loaded. The average age was 58 years old, ranging from 47 to 72 years old. Sample size was not determined, because this study is pilot.

Patients were selected by the following criteria:

1. patients who had been treated by both one‐piece and two‐piece dental implants;
2. dental implants were inserted at least 3 months ago and no more than 12 months ago;
3. dental implants were loaded;
4. dental implants without any signs of inflammation (Gamper et al., 2017):
  - no bleeding on probing,
  - probing depth ≤5 mm,
  - no excessive resorption of marginal bone around dental implant (<2 mm), and
  - no exudate; and
5. patients without any systemic health problems and not using any medication for at least 3 months.

### 2.3 Experimental design and study procedure

Dental implants were divided into three experimental groups:

- Group 1—one‐piece dental implants (ROOTT, TRATE AG);
- Group 2—two‐piece dental implants (multiple manufacturers); and
- control group—healthy teeth.

CS 9000 3D System (Carestream Dental, LCC) was used to take new panoramic radiograph images for the patients. Images were used to compare with before implant surgery and before control visits taken radiographs to determine a loss of marginal bone. If marginal bone resorption was observed, patient was excluded from the study due to selective criteria.

### 2.4 pH measurement equipment and procedure

pH of PICF was measured in the morning (between 9 a.m. and 12 p.m.) using glass microelectrode (Thermo Fisher Scientific™ Orion™ 9863BN Micro pH Electrode) with the measurement accuracy of 0.02 and pH meter (Thermo Fisher Scientific Orion Star™ A121 pH Portable Meter) that was connected to the microelectrode. Before every measurement, electrode was calibrated in three different solutions (pH = 4, pH = 7, and pH = 10).

Before inserting microelectrode into peri‐implant sulcus, the surrounding area was dried with cotton roll. The microelectrode was inserted in peri‐implant sulcus no deeper than 1–1.5 mm and kept there until pH value on pH meter screen is stable. Every measurement was performed three times in 5‐min intervals. In case of bleeding, implant was excluded from the study. pH measurement was also performed around healthy natural teeth, which followed these criteria:

- no bleeding on probing;
- probing depth ≤3 mm;
- no marginal bone resorption; and
- no exudate.

After every measurement, electrode was disinfected using mikrozid disinfectant by Schülke & Mayr, Germany.

### 2.5 Data analysis

Statistical analysis was performed using SPSS 17.0 program. Statistical analysis was performed using SPSS 17.0 program. According to descriptive data analysis, the mean of quantitative variables with a deviation and median with minimal and maximal value was presented. The Kolmogorov–Smirnov and Shapiro–Wilk test was used in the investigation of hypotheses about the normality of the parameter distribution. Mann–Whitney *U* test was applied to compare quantitative sizes two groups and Kruskal–Wallis test of three independent groups. Wilcoxon nonparametric test was used for quantitative‐dependent data. Differences were considered as statistically significant when *P* < 0.05.

The interdependence of qualitative characteristics was evaluated with the help of chi‐squared (*χ* ^2^) criterion.

## 3 RESULTS

Twenty‐seven people participated in the study. The average age of participants was 59.1 (*SD* = 6.3, range 47–72 years old, median—58.0 years old). In total, 58 dental implants (29 one‐piece and 29 two‐piece) and 29 natural teeth were investigated (Table 1). All two‐piece dental implants evaluated in the study were bone‐level implants, and all one‐piece dental implants were tissue‐level implants.

**Table 1:** Demographic data of the study

Title	Female	Male	*P* value
No. of participants/tooth	15/17	8/12	
Age (years), median [min–max]	61.0/62.0 [49–72]	57.0/56.5 [47–66]	0.101/0.066*
Type, *n* (%)			
One‐piece dental implants	15 (31.9)/20 (32.8)	10 (33.3)/15 (32.6)	*χ* ^2^ = 0.139/0.933, *df* = 2, *P* = 0.933/0.995**
Two‐piece dental implants	16 (34.0)/19 (31.1)	9 (30.0)/14 (30.4)	
Natural teeth	16 (34.0)/22 (36.1)	11 (36.7)/17 (37.0)	

*Note*. *df*: degrees of freedom.

* *P* value by Mann–Whitney *U* test.

** *P* value by chi‐squared test.

### 3.1 Mean pH values of PICF and GCF

**Figure 1:**
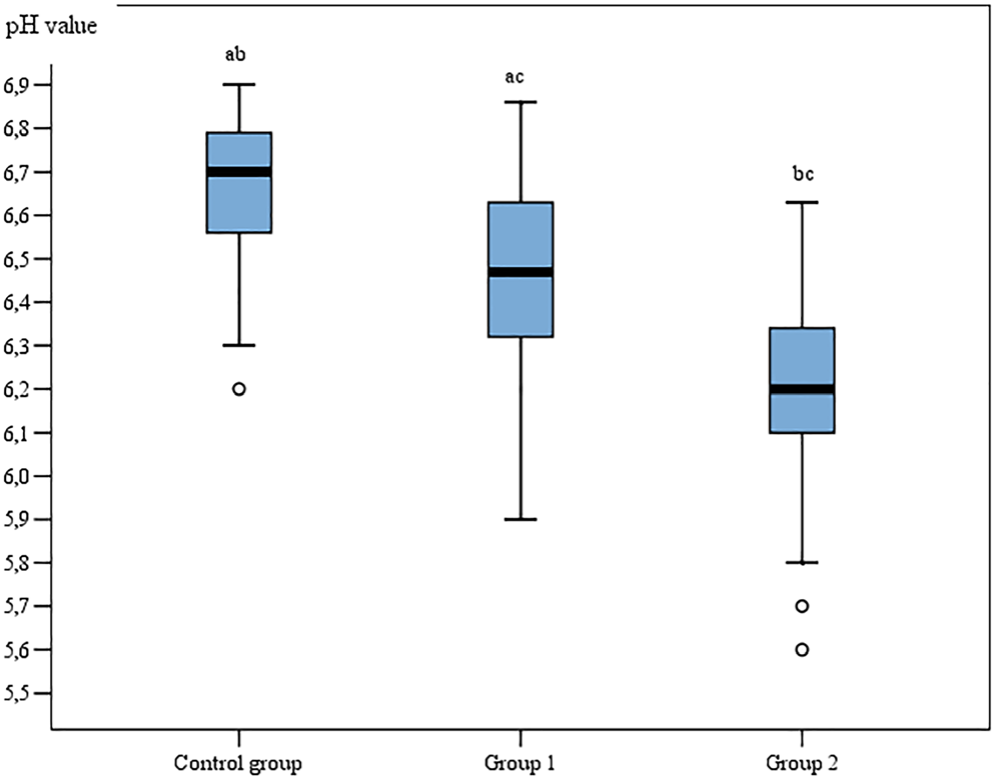
Box plot pH values of peri‐implant crevicular fluid and gingival crevicular fluid, *P* < 0.001. Different letters above bars indicate a significant difference between groups (*P* < 0.05)

The average value of PICF pH statistically significantly differed between Groups 1 and 2 (*P* < 0.001). The average pH value around one‐piece dental implants was 6.46 (value range 5.9–6.86) and around two‐piece dental implants was 6.16 (value range 5.6–6.6). The average pH value of Groups 1 and 2 was lower than in control group (pH = 6.64, value range 6.2–7.0; Figure 1 and Table 2).

**Table 2:** Comparison of pH value characteristics of PICF and GCF

Characteristic	Control group	Group 1	Group 2
*M* (*SD*) Median [min–max]	6.64 (0.19) 6.7 [6.2–6.9]* ^,^ **	6.46 (0.22) 6.47 [5.9–6.86]* ^,^ ***	6.16 (0.24) 6.2 [5.6–6.63]** ^,^ ***
Test of normality	0.168	0.2	0.059
Kolmogorov–Smirnov	0.168	0.2	0.059
Shapiro–Wilk	0.054	0.46	0.341

*Note*. *P* value by Wilcoxon signed‐rank test. GCF: gingival crevicular fluid; *M*: mean; PICF: peri‐implant crevicular fluid; *SD*: standard deviation.

* *P* < 0.05.

** *P* < 0.01.

*** *P* < 0.001.

### 3.2 Correlation between pH and gender

**Figure 2:**
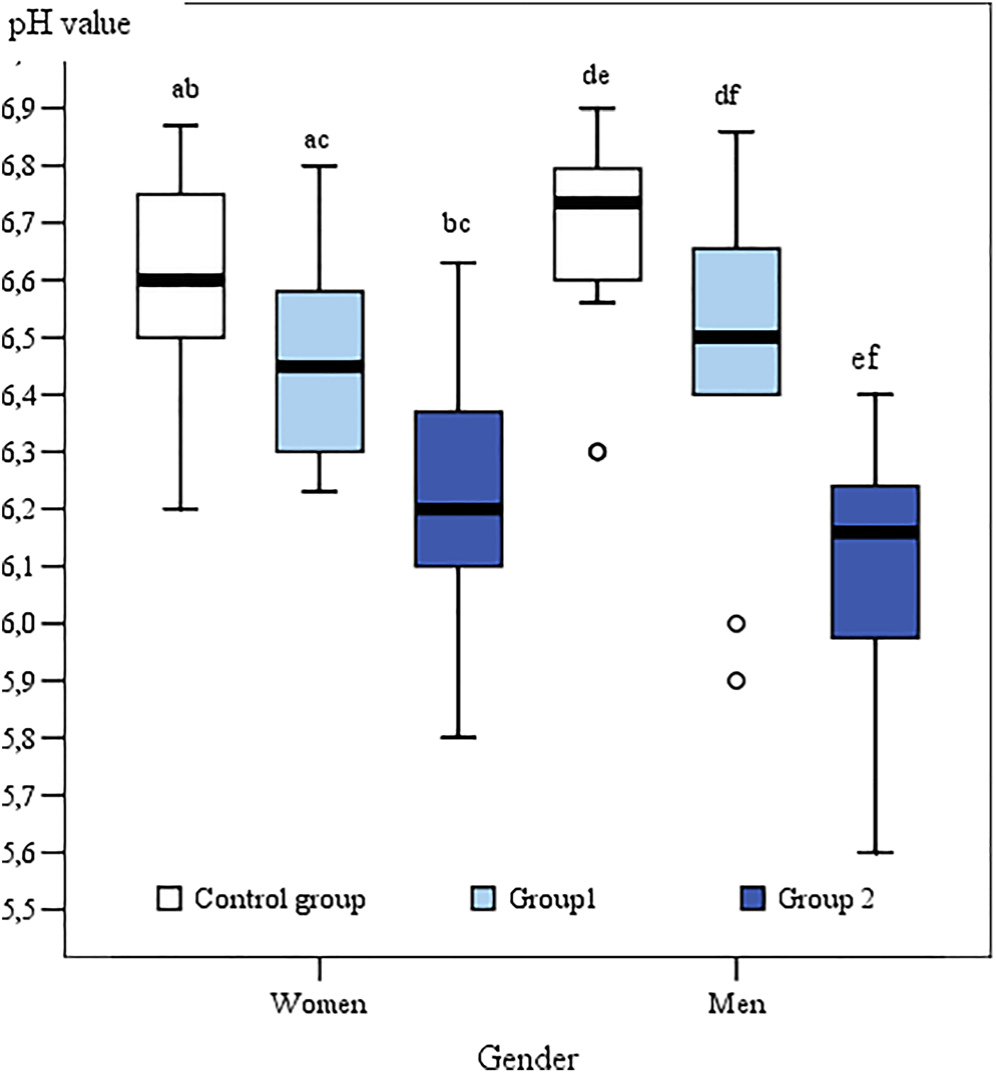
Box plot pH values of peri‐implant crevicular fluid and gingival crevicular fluid in male and female, *P* < 0.05. Different letters above bars indicate a significant difference between groups (*P* < 0.05)

pH of PICF in Group 2 differed between women and men (Mann–Whitney *U* test, *P* < 0.05). In women group, the average pH value of PICF around two‐piece dental implants was 6.21 and in men group was 6.1. In Group 1 and in control group, statistically significant difference was not found (Figure 2 and Table 3).

**Table 3:** Characteristics of pH values of PICF and GCF in male and female

pH value	Female (*n* = 17)	Male (*n* = 12)	*P* value between gender
Control group			
Mean (*SD*) Median [min–max]>	6.62 (0.18)^ab^ 6.6 [6.2–6.87]	6.68 (0.2)^ab^ 6.74 [6.3–6.9]	0.362
Group 1			
Mean (*SD*) Median [min–max]	6.46 (0.18)^ac^ 6.45 [6.23–6.8]	6.47 (0.28)^ac^ 6.5 [5.9–6.86]	0.451
Group 2			
Mean (*SD*) Median [min–max]	6.21 (0.23)^bc^ 6.2 [5.8–6.63]	6.09 (0.25)^bc^ 6.16 [5.6–6.4]	0.277
* *P* value between group	*χ* ^2^ = 21.539, *df* = 2, *P* < 0.001 ^a^ *P* = 0.02, ^b^ *P* < 0.001, ^c^ *P* = 0.003	*χ* ^2^ = 19.234, *df* = 2, *P* < 0.001 ^a^ *P* = 0.043, ^b^ *P* < 0.001, ^c^ *P* = 0.002	

*P* value between gender by Mann–Whitney *U* test. *SD*: standard deviation; *df*: degrees of freedom.

* *P* value between group by Kruskal–Wallis test (multiple comparisons by Mann–Whitney *U* test).

### 3.3 Correlation between pH and age

There was no statistically significant correlation between age and pH values of any group: age and control group: *r* = −0.152, *P* = 0.432; age and Group 1: *r* = 0.036, *P* = 0.855; and age and Group 2: *r* = 0.202, *P* = 0.292.

## 4 DISCUSSION

This clinical study is the first one that investigated pH of PICF around healthy screw‐type dental implants, differing in their design; therefore, we are not able to properly compare our results with other studies. In the study of Nyako et al. (2005), which investigated changes of PICF pH around healthy and failing dental implants, it was found that average pH of PICF around healthy implants is 6.80 (value range 6.35–7.65), whereas around failing implants is 7.20 (value range 5.63–8.50). In this study, the range of PICF pH is narrower, and all values are lower than 7.0. These differences can be explained by different methods used in these studies. Nyako and colleagues used metallic microelectrode in their study, whereas in this study, glass microelectrode was used. Metallic electrode is extremely sensitive for touching surfaces; therefore, in gingival sulcus, it can show almost 1.5 higher pH if it accidentally touches surface of gingiva or tooth/implant. In contrast, glass electrode is sensitive only for H^+^ ions that get into electrode through permeable membrane; therefore, it can register pH value accurately. In addition, there is no information provided about type of implants that Nyako and coworkers investigated in their study. Therefore, it is impossible to properly compare these studies. However, we can compare pH values of GCF received in this study with other studies previously performed. Average pH of GCF (pH = 6.64) in this study endorses other previously performed studies. Borden, Golub, and Kleinberg (1977) show pH of 6.5, Bickel and Cimasoni (1985)—6.9, and Watanabe, Toda, Morishita, and Iwamoto (1982)—6.7.

In this study, a statistically significant difference of pH of PICF was found between two different groups of screw‐type dental implants, differing in design. pH of PICF around one‐piece dental implants was statistically significantly higher than around two‐piece dental implants (*P* < 0.001). This finding can be explained by existence of microgap between implant and abutment in two‐piece dental implants. Even though modern technologies allow to make microgap as narrow as 10 μm, this does not prevent microgap from invasion of microorganisms. The smallest microorganisms that are found in oral cavity are 0.1 μm in size, and their produced toxins, for example, lipopolysaccharides, are even smaller; therefore, they can easily invade a microgap between implant and abutment (Rack et al., 2010). Even eight periopathogenic bacteria species are found among microorganisms that invade microgap, including *Aggregatibacter actinomycetemcomitans* and *Porphyromonas gingivalis*, which are one of the most pathogenic bacteria involved into periodontal diseases. Broggini et al. (2003) found that inflammatory cells are located most densely around microgap and 0.5 mm more coronally. The amount of inflammatory cells was more abundant around two‐piece dental implants comparing with one‐piece dental implants (*P* < 0.001). Early invasion of microorganisms can determine peri‐implant disease and consequently marginal bone resorption. One‐piece dental implants, in contrast, do not have microgap, and consequently, early resorption of marginal bone is very minimal (Östman, Hellman, Albrektsson, & Sennerby, 2007; Parel & Schow, 2005). However, in this study, we did not compare the influence of emergence profile of the crown and type of prosthesis fixation with pH of PICF. Therefore, further investigations are needed.

Günday et al. (2011) also found that wider dental implants have wider peri‐implant sulcus and are characterized by more active production of PICF. For this reason, two‐piece dental implants might be more prone to inflammation of surrounding tissues as they are usually wider than one‐piece implants due to mechanical reasons. Judgar et al. (2014) also discovered that healthy two‐piece dental implants have deeper peri‐implant sulcus than one‐piece dental implants. However, these findings were not statistically significant; therefore, a final conclusion could not be made.

Saliva, whose pH is <7.0, is called acidic and shows pathologic acidity of blood. If this acidemia is chronic, then oral cavity is more prone to caries, halitosis, and periodontitis (Baliga, Muglikar, & Kale, 2013). Takahashi (2005) found out that periopathogenic bacteria grow in slightly acidic environment, and their metabolic products are also slightly acidic: *Fusobacterium* releases glutamic acid and produces acetic and butyric acids. *P. gingivalis*, *Prevotella intermedia*, and *Campylobacter rectus* release succinic acid. Therefore, slightly acidic pH in peri‐implant sulcus is favorable environment for periopathogenic bacteria.

Regardless of the design of screw‐type dental implant, the average pH of PICF differed from the average pH of GCF (*P* < 0.001). The reason for that might be different configuration of periodontal tissues around natural teeth and dental implants. Healthy peri‐implant sulcus is naturally deeper than gingival sulcus. This is explained by the fact that collaged fibers do not attach to the surface of dental implant or abutment; therefore, connective tissue in this location is not formed, only connective epithelium to the marginal bone. Moreover, dental implants do not have periodontal ligament, which is highly vascularized and performs trophic function. For this reason, peri‐implant tissues are less nourished by blood and are more prone to inflammation (Berglundh, Zitzmann, & Donati, 2011).

pH of PICF could also be influenced by material, which dental implants are made from. Although titanium is called one of the most biocompatible and also one of the most corrosion‐resistant metals, however, recently published studies show that titanium oxide membrane dissolves when it is exposed to biologic fluids, such as saliva or PICF, and a surface of dental implant starts corroding. Yu, Addison, Baker, and Davenport (2015) discovered that titanium is especially nonresistant to corrosion, when pH is slightly acidic. Koike and Fujii (2001) also found out that titanium is more prone to corrosion, when oxygen shortage is present in environment. Peri‐implant sulcus meets both of these risk factors, especially around two‐piece dental implants as they usually have deeper sulcus than one‐piece dental implants. Due to corrosion of titanium, dental implants loose volume, and it can be the reason for expansion of microgap between implant and abutment and finally for loosening of crown (Koike & Fujii, 2001). Moreover, it is known that titanium particles cause proinflammatory response in macrophages (Pettersson et al., 2017). Macrophages absorb released titanium particles leading to secretion of inflammatory cytokines, such as interleukin‐1β, interleukin‐6, and tumor necrosis factor‐α, which are strong inducers of osteoclastogenesis and bone resorption (Kudo et al., 2002).

In this clinical study, we found that age can also influence pH of PICF, but statistically significant difference was found only in one‐piece implant group (*P* = 0.004). Moreover, we found that pH of PICF around two‐piece dental implants is more alkaline in women than in men (*P* < 0.001). However, the results of this pilot study are not sufficient to make final conclusions about influence of age and gender to acidity of PICF; therefore, further investigations are much needed.

In this study, we also measured pH of PICF in three male smokers. pH range around one‐piece dental implants was 5.4–5.8 and around two‐piece dental implants was 5.36–5.74. However, sample size was too small to make an experimental group; therefore, these results were not included in this study. Voelker, Simmer‐Beck, Cole, Keeven, and Tira (2013) found out that saliva of smokers is more acidic than of nonsmokers. Even though we are not able to make conclusions, we can predict that smoking plays an important role in pH of PICF as well as GCF.

This is the first study ever performed that investigated the influence of dental implant design for acidity of PICF. Etiology of peri‐implant diseases is very complex; therefore, one diagnostic parameter is not enough to determine accurate diagnosis and level of activity of inflammation. Determination of PICF pH together with already applicable diagnostic methods shows a diagnostic potential in early determination of peri‐implant tissues inflammation. Accurate and quick diagnosis, especially in early stages, is extremely important in order to avoid difficult and not always predictable treatment. Nonsurgical treatment protocol usually only temporarily slows down a progression of inflammation, whereas surgical treatment is complicated for both patient and doctor, and results usually remain unpredictable. Knowing physiologic pH values of PICF, we will continue our investigations in order to find out how acidity of PICF changes during inflammation and if there is a threshold differentiating transition from acute to chronic stage of peri‐implant tissues inflammation.

## 5 CONCLUSIONS

pH of PICF around healthy screw‐type dental implants is slightly acidic and is lower than pH of GCF of healthy natural teeth. Acidity of PICF depends on design of dental implants. One‐piece dental implants show more alkaline pH of PICF than two‐piece dental implants. Determination of PICF pH using glass microelectrode is a suitable method for pH measurement in peri‐implant sulcus and shows a diagnostic potential in determination of peri‐implant diseases.

## CONFLICT OF INTEREST

We confirm that the authors of this study have no conflict of interest relevant to the content of the submission. This research did not receive any specific grant from funding agencies in the public, commercial, or not‐for‐profit sectors.
